# Molecular Behavior of HMGB1 in the Cochlea Following Noise Exposure and *in vitro*

**DOI:** 10.3389/fcell.2021.642946

**Published:** 2021-02-25

**Authors:** Lili Xiao, Yan Sun, Chengqi Liu, Zhong Zheng, Ying Shen, Liang Xia, Guang Yang, Yanmei Feng

**Affiliations:** ^1^Department of Otolaryngology-Head and Neck Surgery, Shanghai Jiao Tong University Affiliated Sixth People’s Hospital, Shanghai, China; ^2^Otolaryngology Institute of Shanghai Jiao Tong University, Shanghai, China; ^3^Shanghai Key Laboratory of Sleep Disordered Breathing, Shanghai, China; ^4^Zhangjiagang TCM Hospital Affiliated to Nanjing University of Chinese Medicine, Zhangjiagang, China

**Keywords:** noise, hearing loss, cochlea, high-mobility group box 1, spatiotemporal distribution

## Abstract

Noise-induced hearing loss (NIHL) is characterized by cellular damage to the inner ear, which is exacerbated by inflammation. High-mobility group box 1 (HMGB1), a representative damage-associated molecular pattern (DAMP), acts as a mediator of inflammation or an intercellular messenger according to its cellular localization. Blocking or regulating HMGB1 offers an attractive approach in ameliorating NIHL. However, the precise therapeutic intervention must be based on a deeper understanding of its dynamic molecular distribution and function in cochlear pathogenesis after acoustic trauma. Here, we have presented the spatiotemporal dynamics of the expression of HMGB1, exhibiting distribution variability in specific cochlear regions and cells following noise exposure. After gene manipulation, we further investigated the characteristics of cellular HMGB1 in HEI-OC1 cells. The higher cell viability observed in the HMGB1 knocked-down group after stimulation with H_2_O_2_ indicated the possible negative effect of HMGB1 on cellular lifespan. In conclusion, this study demonstrated that HMGB1 is involved in NIHL pathogenesis and its molecular biology has essential and subtle influences, preserving a translational potential for pharmacological intervention.

## Introduction

Hearing loss is often induced by loss of sensory hair cells (HCs) in the inner ear cochlea, which functions in the transduction of sound waves into electric signals (Zhu et al., 2018; Liu et al., 2019; Qi et al., 2020). WHO reported that 466 million people suffer from hearing loss worldwide, because of genetic factors, infectious diseases, chronic cochlear infections, aging, exposure to noise, and ototoxic drugs (Gao et al., 2019; Zhang et al., 2019; He et al., 2020). Noise-induced hearing loss (NIHL) is the most common type of sensorineural hearing loss, ranking first among occupational diseases, seriously affecting the lives of patients, and greatly increasing the social burden (Carroll et al., 2017; Murphy et al., 2018). The mechanisms underlying noise-induced damage to critical cochlear cells have been reported to include oxidative damage, mechanical shearing forces, and glutamate excitotoxicity (Zhou et al., 2020), which eventually induce apoptotic cell death in HCs, especially the outer HCs of the basal turn (Sun et al., 2014; Yu et al., 2017; Li et al., 2018a; Zhang et al., 2019).

In recent years, increasing evidence has demonstrated that NIHL could be exacerbated by inflammation of the cochlea (Kalinec et al., 2017; Wood and Zuo, 2017; Hu et al., 2018; Frye et al., 2019). Resident cochlear cells, subsequently infiltrating immune cells, and released cytokines or reactive oxygen species (ROS) have been suggested to all interact toward a positive or negative effect in cochlear pathogenesis (He et al., 2016; Lang et al., 2016; Wood and Zuo, 2017; Hu et al., 2018). Specifically, damage-associated molecular patterns (DAMPs), the cellular byproducts of damage, are known to play an essential role in this inflammation. They could stimulate pattern recognition receptors, leading to the rapid release of proinflammatory cytokines and production of ROS, thus insulting sensorineural cells (Tang et al., 2012; He et al., 2017; Wood and Zuo, 2017). However, the type and function of DAMPs in the cochlea following acoustic trauma is rarely clear.

More specifically, DAMPs include pathogen-associated molecular patterns (PAMPs) and “alarmins,” endogenous molecules that signal tissue and cellular damage. The high-mobility group box 1 (HMGB1) protein, a representative alarmin, is known to act as a mediator of inflammation or an intercellular messenger when it reaches the extracellular environment (Bianchi et al., 2017). Extracellular HMGB1 has been shown to be typically involved in various immune responses, such as neuroimmune, chemokine−and cytokine−like activity via recently elucidated signal, and molecular transport mechanisms (Yang et al., 2013; Bianchi et al., 2017). Blocking the release or activation of extracellular HMGB1 has been proposed as a promising therapeutic target for inflammatory diseases. Due to its relatively independent function accompanying its different cellular localization, a deeper understanding of HMGB1 biology in the cochlea is indispensable for guiding future precise therapeutic interventions. However, the spatiotemporal expression of HMGB1 in cochlea with acoustic injury has not been systemically investigated.

Until recently, the molecular biology of HMGB1, especially it signaling and role in inflammation, has been extensively studied and broadly recognized. Of the receptors important for the activity of HMGB1, the toll-like receptor (TLR)-4 and receptor for advanced glycation end products (RAGE) have been reported to be the 2 critical interacting receptors leading to the upregulation of proinflammatory cytokines via the NF-κB and MAP kinase signaling pathways (Andersson and Tracey, 2011; Harris et al., 2012; Ott et al., 2014; Yang et al., 2015a; Paudel et al., 2019). Correspondingly, our recent study demonstrated that the activation of MAPK and NF-κB could facilitate the morphological damage and dysfunction of inner ear tissues during the immune response (Xia et al., 2019). Besides, Shih et al. showed that noise-induced increasing level of HMGB1 in the spiral ligament was significantly reduced in the group given dexamethasone in the middle ear compared with the control group, suggesting that HMGB1 participated in noise-induced cochlear inflammation (Shih et al., 2019). Overall, HMGB1 could be a potentially significant candidate as the initial factor alarming for cochlear damage due to its unique biology and role in immune events. Furthermore, newborn HMGB1-knockout mice were shown to die of hypoglycemia within 24 h, suggesting its important role in gene transcription. However, the effects on transcription were cell-type specific because cell lines lacking HMGB1 have been reported to be able to survive *in vitro* (Calogero et al., 1999). Therefore, gene manipulation could be a possible option for further exploring the function of HMGB1 in specific cochlear cell lines.

In the present study, we investigated the spatiotemporal dynamic expression of HMGB1 in the cochlea following noise exposure (NE), which showed a variable pattern of distribution in specific cochlear regions. The cellular biology of HMGB1 in HEI-OC1 cells was also explored in an oxidative stress model following treatment with H_2_O_2_, indicating the positive impact of a HMGB1-knockdown on the cellular lifespan. Therefore, selectively targeting HMGB1 at a specific cochlear region with the consideration of temporal factors would offer a novel perspective on the specific intervention for NIHL in the future.

## Materials and Methods

### Animals

A total of 42 male C57BL/6 mice at 4–5 weeks of age were purchased from the Shanghai Songlian Lab Animal Field (Shanghai, China). Animals were maintained in a temperature-controlled room under a 12-h light cycle and were acclimated for at least 1 week before the experiments. All mice were housed in individually ventilated cages (5 per cage) and given access to free water and food. Animals were subjected to auditory brainstem response (ABR) measurements before exposure to ensure they have a normal ABR threshold. Samples from 30 mice (six mice per group) were used for western blotting, while samples from 12 mice (three mice per group) were used for fluorescence immunocytochemical investigations. The cochleae of each side were collected for cryostat sectioning and basilar membrane dissection, respectively. All research protocols involving the use and care of animals were approved by the Ethics Committee of the Shanghai Jiao Tong University Affiliated Sixth People’s Hospital (permit number DWLL2017-0295).

### Noise Exposure

Awake mice were exposed to white noise high-pass filtered at 4 kHz and 110 dB SPL for 2 h. The noise signal was released from a loudspeaker with an upper-frequency limit of 22 kHz (Pyramid TW67 Super tweeters, Pyramid, Brooklyn, NY, United States). Generally, four mice (one mouse per metal wire cage, 10 cm × 10 cm × 15 cm) were located in the center of the sound chamber, in which animals were left unrestrained.

### Electrophysiological Evaluation

The ABR to tone burst (TB) at different frequencies was evaluated for all mice before and 1-, 3-, 7-, and 14-days after NE. Animals were lightly anesthetized with an intraperitoneal injection of 1% sodium pentobarbital (75 mg/kg), and their body temperature was maintained at 37°C with a heating pad. Acoustic stimuli were delivered using an electrostatic speaker (Fostex FT28D Dome Tweeter; Madisound, Middleton, WI, United States), positioned 10 cm in front of the mouse head. The ABR was recorded with three subdermal electrodes: the recording electrode was inserted at the vertex of the skull, while the reference and grounding electrodes were positioned separately on the mastoid region of the two sides. The biological signals collected by the electrodes were led to a RA4PA preamplifier from Tucker-Davis Technologies (TDT System III; Alachua, FL, United States). Stimulus generation and biosignal acquisition were conducted processed by using the RZ6 BioAMP Processor (TDT System III; Tucker-Davis Technologies, Alachua, FL, United States). Parameters were similar to those used in our previous study (Fan et al., 2020; Zhang et al., 2020). Briefly, the signal of acoustic stimuli is TB for 10 ms at 0.5 ms rise/fall time, and 21.1/s stimulation rate, starting from 90 to 0 dB SPL in decreasing steps of 5 or 10 dB. The ABR threshold was defined as the lowest level of stimulus that yielded a repeatable waveform.

### Cell Culture and Hydrogen Peroxide Treatment of HEI-OC1 Cells

HEI-OC1, an inner ear cell line, was purchased from UCLA Technology Development Group (Los Angeles, CA, United States) and cultivated in Dulbecco’s modified Eagle’s medium (Gibco, United States) supplemented with 10% fetal bovine serum (FBS) (Performance Plus; 16000-044; Gibco) and 10 mg/mL ampicillin sodium solution (sterile) (B540722; Sangon Biotech, Shanghai, China) at 33°C in a humidified 5% CO_2_ atmosphere. After 24 h, cells were grown to 70–80% confluency, and the regular medium was exchanged for medium with or without hydrogen peroxide.

### Morphology

Mice were anesthetized and decapitated following the ABR recording at the end point. Both the left and right temporal bones were carefully removed, and the bulla was opened to expose the cochlea. The cochleae were perfused locally with a solution of 4% paraformaldehyde in PBS (pH 7.4) and kept in this fixative for 2 h at 4°C, then rinsed with PBS, and decalcified in 10% sodium EDTA solution (adjusted with HCl to pH 7.4) overnight at 4°C. The cochlea of one side was dissected under a microscope, whereas that of the other side was immersed in 15% sucrose solution for 2 h, followed by overnight immersion in sucrose (30%) at 4°C. Cochleae were embedded in optimal cutting temperature compound (OCT), frozen at −80°C, and cryosectioned at a thickness of 12 μm using a cryostat microtome (Leica CM1860, Leica Biosystems Nussloch GmbH, Nussloch, Germany). Sections were processed at the same time for all staining procedures. All slides and the basilar membrane were washed with PBS, incubated in 1% TritonX-100 for 1 h, and immersed in 5% goat serum for 1 h at 25°C and then in primary antibodies at 4°C overnight. Primary antibodies used included: monoclonal rabbit anti-HMGB1 Ig-G (1:200; ab79823, Abcam) and monoclonal mouse anti-beta III tubulin Ig-G (1:200; ab78078, Abcam). Subsequently, they were incubated with either Alexa-Fluor-488 goat anti-rabbit Ig-G (H + L) (1:500; Invitrogen, Carlsbad, CA, United States) or Alexa-Fluor-555 goat anti-mouse Ig-G (H + L) (1:500; Invitrogen) at 25°C for 2 h in darkness, followed by incubation with DAPI (ab104139, Abcam) for 5 min, and then their edges were sealed with nail polish.

Cells were cultured in coverslips and subjected to different treatments. After removing the culture medium, cells were rinsed twice with PBS, fixed with 4% paraformaldehyde for 15–20 min, and then permeabilized with 0.5% TritonX-100 for 40 min. Subsequently, cells were blocked with 5% normal goat serum for 1 h at 25°C, followed by incubation with primary HMGB1 rabbit monoclonal antibody (1:200 in PBS; Abcam) overnight at 4°C. After three washes in PBS, cells were incubated in the dark with Alexa Fluor 488-conjugated goat anti-rabbit IgG (H + L) (1:500 in PBS; Invitrogen) for 2 h at 25°C. After four washes with PBS (30 min each), cells were also stained with Phalloidin iFlour 555 reagent (1:5,000 in PBS; Abcam) for 10 min, to mark cell structure. After rinsing in PBS, specimens were immersed for 5 min in fresh DAPI solution in the dark to label the nuclei of cells. Immunocytochemistry images of basal turn in each cochlear sample and HEI-OC1 cells were taken under a 20× and 63× magnification lens respectively with identical Z-stack conditions using a confocal laser-scanning microscope (LSM 710 META; Zeiss, Shanghai, China) and analyzed using the ZEN 2011 software (Zeiss, Oberkochen, Germany).

### Cell Vitality Assay

Cultured HEI-OC1 cells were plated in each well of a 96-well plate in five copies. After attachment, cells were treated with a gradient concentration of H_2_O_2_ (0.1, 0.2, 0.5, and 1.0 mM) for 4, 8, 12, and 24 h. Following treatment, cells were incubated for 2 h with Cell Counting Kit 8 (CCK-8) reagent (100 μL/mL, MCE, Shanghai, China) at 37°C for 2 h, according to the manufacturer’s protocol. Absorbance was measured at 450 nm using a microplate reader (Bio-Rad), with results being normalized to untreated blank control cells (100%).

### Small Interfering RNA Gene Silencing

The expression of HMGB1 was knocked down by transfection with candidate HMGB1 small interfering RNA (siRNA) (DNABIO; Shanghai, China). Cells were seeded at 2 × 10^5^ cells per well in a six-well plate the day before transfection. Cells were transfected in 2 mL of culture medium containing 5 μL of HMGB1 siRNA or non-targeting control siRNA using Lipofectamine 3,000 in Opti-MEM medium (Invitrogen, Carlsbad, CA, United States), following the manufacturer’s protocol. After 48 h of transfection, cells were treated as designated. The sequence of siRNA-1 targeting of the mice HMGB1 gene was sense 5′-CCUCAUAUGCAUUCUUUGUTT-3′ and antisense 5′-ACAAAGAAUGCAUAUGAGGAC-3′. The sequence of siRNA-2 was sense 5′-GCUGAAAAGAGCAAGAAAATT-3′ and antisense 5′-UUUUCUUGCUCUUUUCAGCCT-3′.

### Western Blotting

The total protein of cell and cochlea samples was extracted using RIPA lysis buffer (EpiZyme; Shanghai; China). The concentration of each protein sample was quantified using a BCA protein assay kit (EpiZyme). Protein samples (30–40 μg) were subjected to 12.5% polyacrylamide gel electrophoresis and then transferred onto a nitrocellulose membrane (Pierce) and blocked with 5% non-fat milk in EZ Buffer H 1 × TBST Buffer (Sangon Biotech; Shanghai, China) for 1 h. The membranes were washed and then incubated with various primary antibodies (1:30,000 rabbit monoclonal anti-HMGB1, Abcam; 1:1,000 rabbit polyclonal anti-caspase 3, CST) at 4°C overnight. After three washes with TBST (1×), membranes were incubated with appropriate secondary antibodies (1:5,000) for 2 h at 25°C. Following extensive rinsing of membranes, the immunoreactive bands were detected using a chemiluminescence imaging system (Bio-Rad). GAPDH (CST, 1:3,000) was used as a sample loading internal control. Bands were quantified using the ImageJ software (National Institutes of Health, Bethesda, MD, United States).

### Statistical Analysis

All data were expressed as the mean ± SD. ANOVAs followed by *post hoc* testing (Holm-Sidak’s method) were performed using SigmaPlot (ver. 14; Systat Software Inc., San Jose, CA, United States). In all analyses, a value of *p* < 0.05 was considered statistically significant.

## Results

### Shift of ABR Threshold and Level of HMGB1

**FIGURE 1:**
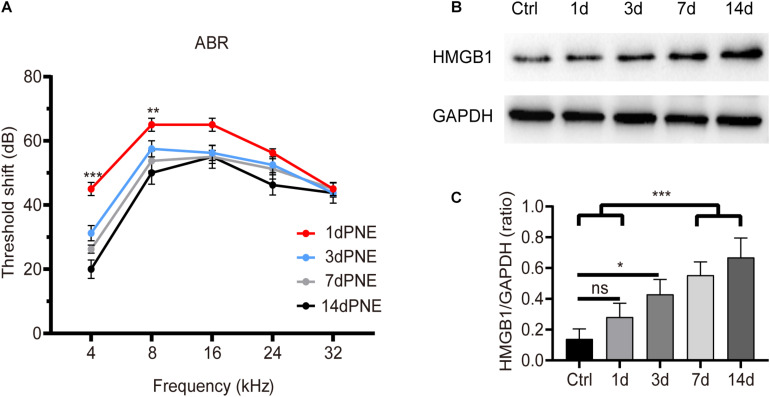
Shift of ABR threshold and total cochlear level of HMGB1 at different time points after noise exposure. **(A)** Threshold shifts (±SD) at 1, 3, 7, and 14 day-PNE (4–22 kHz broadband noise at 110 dB SPL for 2 h). **(B)** Gradual increase in the expression of HMGB1 in the cochlea after noise exposure. **(C)** Densitometry analysis of total HMGB1 band densities normalized to GAPDH confirmed a significant increase in HMGB1 after noise trauma. Data **(C)** is presented as means ± SD, *n* = 3 in each group. PNE, post-noise exposure. *p < 0.05, **p < 0.01, and ***p < 0.001 were significant levels of the difference between groups; “ns”, no significant.

Figure 1 shows the shifts in the ABR threshold and the expression of HMGB1 in the cochlea at different time points after NE. For the ABR result (Figure 1A), two-way ANOVA followed by *post hoc* tests were performed for each group. We observed a significant threshold shift as demonstrated by the effect in time points after NE (*F*_3_,_60_ = 21.214, *p* < 0.001). *Post hoc* pairwise tests showed a significant elevation of the ABR threshold shift at 4 kHz in the 1-day post-noise exposure (1dPNE) group as compared with those in the 3dPNE, 7dPNE, and 14dPNE groups (*t* = 4.286, 6.231, 9.710, respectively, *p* < 0.001), as well as at 8 kHz with only the 14dPNE group (*t* = 3.109, *p* = 0.017).

Correspondingly, we evaluated the level of total cochlear HMGB1 using western blot analysis and revealed its significance for each group by performing one-way ANOVA (*F*_4_,_10_ = 14.622, *p* < 0.001). *Post hoc* pairwise tests showed a significant increase in the 7dPNE and 14dPNE groups compared with the control (*t* = 5.324, *p* = 0.003; *t* = 3.45, *p* = 0.037, respectively) and 1dPNE (*t* = 6.815, *p* < 0.001; *t* = 4.941, *p* = 0.005, respectively) groups. We found that the level of HMGB1 increased significantly in the 3dPNE group (*t* = 3.706, *p* = 0.028) but not in the 1dPNE group (*t* = 1.874, *p* = 0.315) compared with the control group. Furthermore, no significance was demonstrated across the 3dPNE, 7dPNE, and 14dPNE groups, suggesting that the level of total cochlear HMGB1 was gradually increased toward a plateau 3 days after NE.

### Cellular Expression of HMGB1 in the Basilar Membrane

**FIGURE 2:**
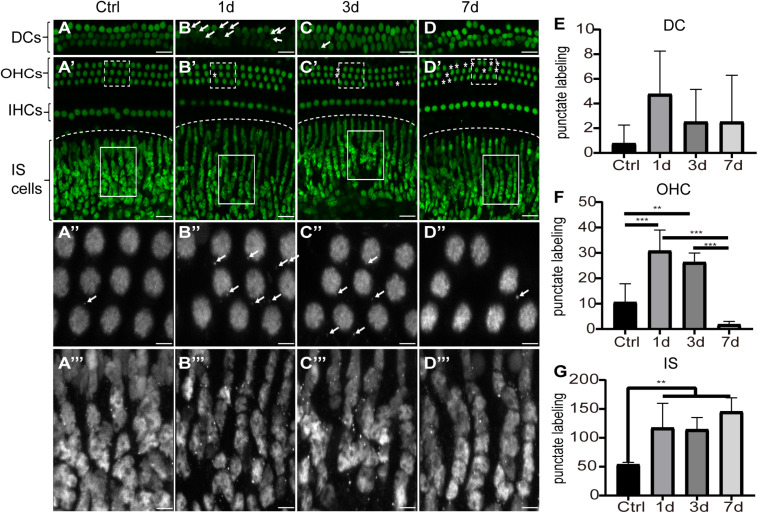
Distribution of optical density of HMGB1 labeling in the basal turn from the cochlear basilar membrane. **(A–D)** Representative images of HMGB1 immunostaining at different confocal levels in the control, 1dPNE, 3dPNE, and 7dPNE groups, respectively. **(E–G)** Quantification of punctate labeling in DCs, OHC, and IS, respectively. White arrows point to the optical HMGB1-immunostained particles. Asterisks represent missed OHCs. Square regions with dash and solid line are magnified below. DCs, Deiters cells; OHCs, outer hair cells; IHCs, inner hair cells; IS cells, inner sulcus cells. All data are presented as means ± SD, *n* = 3 in each group. Scale bars 20 μm **(A–D,A′–D′)**, 5 μm **(A′′–D′′,A′′′–D′′′)**. ^∗∗^*p* < 0.01, ^∗∗∗^*p* < 0.001.

To investigate the cellular expression of HMGB1 in the cochlea, we performed immunofluorescence experiments. We detected the strong dense optical HMGB1 labeling in the nuclei of all cells examined in this study. Figure 2 showed distribution of optical density of HMGB1 labeling in the basal turn from the cochlear basilar membrane at different confocal levels in the control, 1dPNE, 3dPNE, and 7dPNE groups. The absence of outer hair cells (OHCs) presented in Figures 2B′–D′ indicated an irreversible damage caused by NE. Correspondingly, we noted that the arrangement of Deiters cells (DCs) seemed to be disordered in the 7dPNE group because of the increased absence of OHCs. In this study, we mainly focused on several specific confocal levels of immunostaining.

We observed the punctuate labeling of extranuclear HMGB1 in the basilar membrane of all groups, but with a variety of distribution at different confocal levels. We also evaluated the number of punctate labeling in three regions: DCs, OHCs, and inner sulcus (IS) cells. Using one-way ANOVA, we overall detected significant differences in the OHC (*F*_3_,_12_ = 21.26, *p* < 0.001) and IS cell (*F*_3_,_12_ = 8.314, *p* = 0.003) regions, but not in the DC region (*F*_3_,_12_ = 1.2, *p* = 0.352) across groups. We further found that at the OHC level, the number of punctate HMGB1 labeling was significantly increased in the 1dPNE (*t* = 4.892, *p* = 0.001) and 3dPNE (*t* = 3.805, *p* = 0.008) groups compared with the control group. In contrast, it was drastically decreased in the 7dPNE group. When compared with the control group, the number of punctate labeling at the IS level was demonstrated to be significantly elevated following NE at the 1dPNE (*t* = 3.358, *p* = 0.02), 3dPNE (*t* = 3.213, *p* = 0.029), and 7dPNE (*t* = 4.389, *p* = 0.002) groups.

**FIGURE 3:**
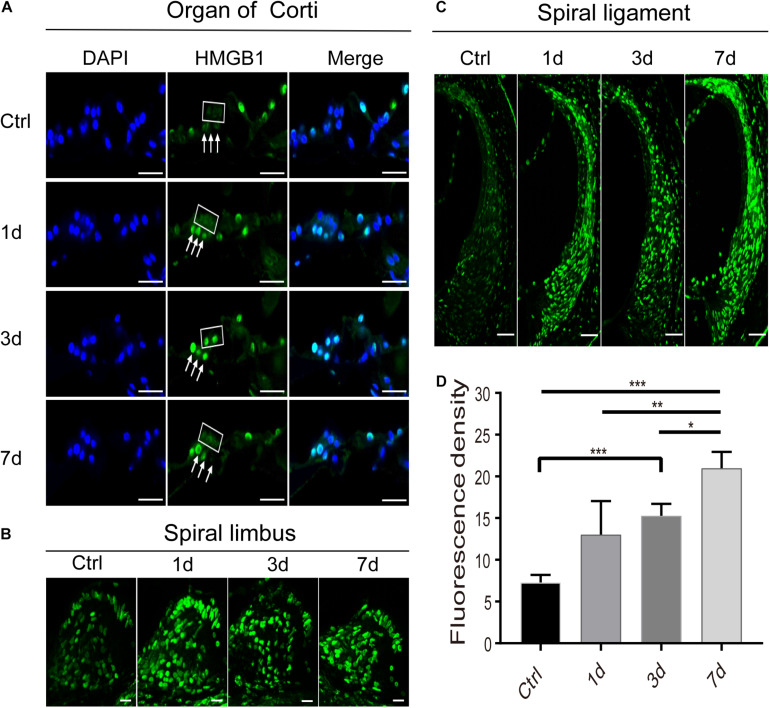
Spatiotemporal dynamics of HMGB1 at the organ of Corti **(A)**, spiral limbus **(B)**, and spiral ligament **(C)** in the basal turn of cochlea after acoustic trauma. The square and arrows in panel **(A)** identify the region of outer hair cells and Deiters cells, respectively. **(D)** Quantitative measurement of average fluorescence density (AFD) in the spiral ligament of panel **(C)**. Data is presented as means ± SD, *n* = 3 in each group. Scale bars 50 μm **(C)**, 20 μm **(A,B)**. *p < 0.05, **p < 0.01, and ***p < 0.001.

We then investigated the pattern of expression of HMGB1 in the region of OHC and DCs after NE. Interestingly, we observed a specific expression pattern of HMGB1 in both OHC and DCs, as demonstrated in images obtained with the same confocal parameters of samples prepared with the same process (Figure 3A). We found that the level of HMGB1 in the OHC region was not evident in the control group, whereas it was shown to subsequently increase to a predominate level at 3dPNE (Figure 3A). Regarding DCs, we noted that the dense labeling of HMGB1 was gradually increased toward a plateau at 3dPNE but was then relatively decreased at 7dPNE.

### Expression of HMGB1 in the Non-organ of Corti Tissues

**FIGURE 4:**
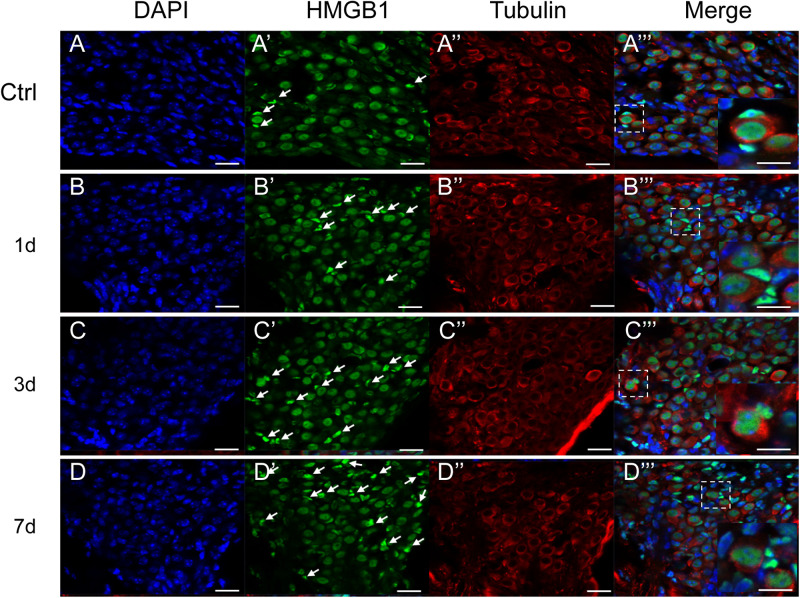
Representative images of immunostaining against HMGB1 (Green) in the SGN region. All images were taken from the cochlear basal turn. White arrows point to glial cells surrounding SGNs with evidently higher density of optical HMGB1. The square region in the merged picture depicts the distribution of HMGB1 in SGNs and glial cells, and is magnified at the right corner. The cytoplasm of SGNs was specifically labeled for tubulin (red), and nuclei were labeled with DAPI (blue). Scale bars 20 μm **(A–D,A′–D′,A′′–D′′)**, 10 μm **(A′′′–D′′′)**.

Apart from the region of the organ of Corti, we also observed and compared the distinctive expression of HMGB1 in the spiral limbus (Figure 3B), spiral ligament (Figure 3C), and spiral ganglion cells (SGNs) region (Figure 4) at different time points after acoustic trauma. We observed a higher optical labeling density in both the spiral limbus and spiral ligament after NE. Furthermore, we quantitatively measured the average fluorescence density (AFD) of HMGB1 in the spiral ligament region due to its possible essential role in the inflammatory response. Accordingly, using one-way ANOVA we demonstrated a significant difference across groups (*F*_3_,_8_ = 16.966, *p* < 0.001). A significant elevation of AFD was observed in the 7dPNE group when compared with the AFD of the control (*t* = 7.039, *p* < 0.001), 1dPNE (*t* = 4.082, *p* = 0.014), and 3dPNE (*t* = 2.919, *p* = 0.038), respectively. In addition, the AFD of the 3dPNE group was also shown to be significantly higher than that of the control group (*t* = 4.12, *p* = 0.017).

We did not observe any evidently increased optical density of HMGB1 in the SGN region, across all groups (Figure 4). By specifically labeling the cytoplasm of SGNs for tubulin (red), we could distinguish SGNs and identify their borders from surrounding cells, such as glial cells, as indicated in previous studies (Fang et al., 2018; Liu et al., 2020). The expression of HMGB1 in SGNs was shown to be mainly located in the nucleus and not in the cytoplasm, consistent with two previous reports but contradicting to the results of another study (Ladrech et al., 2017; Fang et al., 2018; Liu et al., 2020). Intriguingly, the proportion of glial cells with a high density of optical HMGB1 was shown to be increased in a time interval-dependent manner post-NE, peaking in the 7dPNE group (white arrows pointed in Figure 4). Moreover, the magnified view in the right corner of the merged image shows the possible evolution of the spatial expression of HMGB1 between SGNs and glial cells. We further noted the continuity of HMGB1 immunostaining across SGNs and glial cells in the 3dPNE group, indicating a possible translocation of HMGB1 across them (Figure 4C′′′).

### Impact of HMGB1 Knockdown on the Viability of HEI-OC1 Cells

**FIGURE 5:**
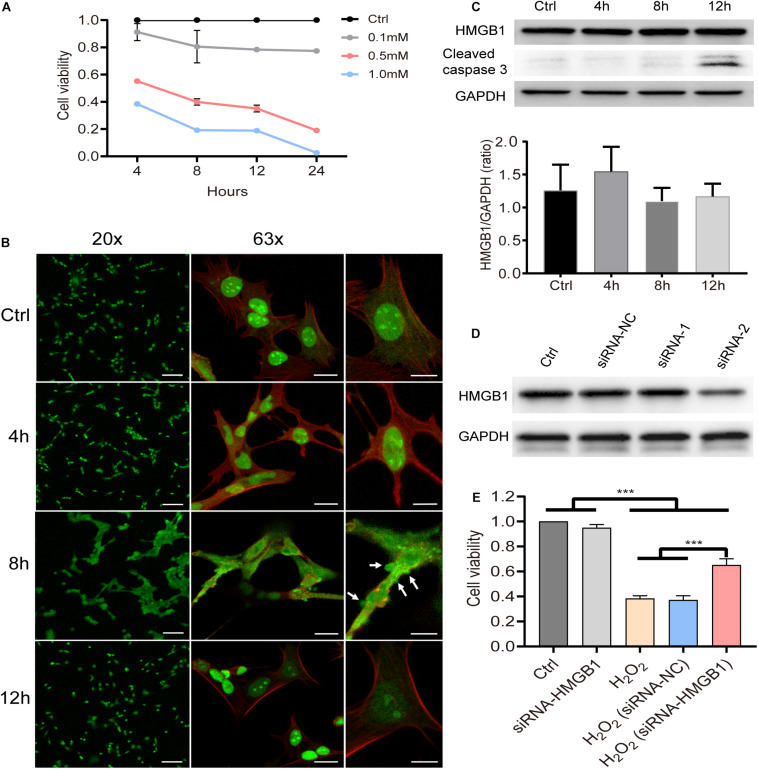
Molecular behavior of HMGB1 and its impact on cell viability *in vitro*. **(A)** Cell viability of HEI-OC1 at 4, 8, 12, and 24 h after stimulation with gradient concentration (0.1, 0.5, and 1 mM) of H_2_O_2_. Data **(A)** is presented as means ± SD, *n* = 3 in each group. **(B)** Representative images of immunostaining against HMGB1 from low to high field of view of samples pretreated with 0.5 mM H_2_O_2_ (green: HMGB1; red: phalloidin). **(C)** Level of HMGB1 and caspase-3 at different time points in cells treated with 0.5 mM H_2_O_2_. Data **(C)** is presented as means ± SD, *n* = 3 in each group. **(D)** Screening result of two candidate siRNAs on knocking down HMGB1. **(E)** Impact of HMGB1 knockdown on the viability of HEI-OC1 cells 8 h after treatment with H_2_O_2_ (0.5 mM). Data **(E)** is presented as means ± SD, *n* = 6 in each group. Scale bars 100 μm (**B** left panel), 20 μm (**B** middle panel), 10 μm (**B** right panel). ***p < 0.001.

The HEI-OC1 cell line is known to preserve the specific cell biology of OHCs and it was employed here to explore the underlying molecular mechanisms after acoustic trauma using a gene manipulation method. First, we confirmed the viability of HEI-OC1 cells using the CCK8 test at different time points after stimulation with different concentrations of H_2_O_2_ (Figure 5A). Based on these, 0.5 mM H_2_O_2_ was identified as the appropriate condition in the following experiments. We accordingly evaluated the level of HMGB1 at 4, 8, and 12 h after treatment with H_2_O_2_. We performed a one-way ANOVA followed by *post hoc* tests for each group and found that no significant intergroup differences were observed (*F*_3_,_8_ = 1.254, *p* = 0.353).

As can be observed in the immunostaining image (Figure 5B), however, HMGB1 was found to be extensively translocated from the nucleus to the cytoplasm 8 h after stimulation with H_2_O_2_. The edge of the cell membrane outlined by staining with phalloidin indicated that HMGB1 might be released from the cell before cell disintegration. More importantly, it is worth noting that the overall changes in cell morphology were shown to be quite different at this time. We observed varying degrees of vesicular-like budding that might have been related to the apoptosis or death process in HEI-OC1 cells. Interestingly, at 12 h, most cells were demonstrated to revert to their typical structure, with HMGB1 being redistributed within the nucleus.

Using western blotting, we identified target siRNAs for further knockdown of HMGB1 (Figure 5D). Subsequently, we investigated the effect of HMGB1 knockdown on cell viability in the HEI-OC1 oxidative stress model (Figure 5E). We found that compared with the control group and the siRNA-HMGB1 group, the cell viability in groups pretreated with H_2_O_2_ was significantly reduced (*p* < 0.001). However, the group with the HMGB1 knockdown was demonstrated to significantly preserve a higher cell viability compared with the H_2_O_2_ and the H_2_O_2_ (siRNA-negative control) groups (*p* < 0.001).

## Discussion

Here, we demonstrated the spatiotemporal dynamics of the expression of HMGB1 in the cochlea after NE. We found that the total level of cochlear HMGB1 was gradually increased following NE, exhibiting however a distribution variability in specific cochlear regions and cells. Specifically, noise induced a robust accumulation of HMGB1 in the spiral ligament, but not in the region of SGNs, which coincided with the cochlear level as tested by western blotting. In contrast, despite the extranuclear punctate labeling for HMGB1 revealing a varying distribution, we observed that HMGB1 was primarily located in the nuclei of cochlear cells. In addition, we noted the occurrence of a temporary translocation of HMGB1 from the nucleus to the cytoplasm as well as the extracellular space, in HEI-OC1 cells 8 h after treatment with H_2_O_2_. However, nuclear retention of HMGB1 was shown to recur at 12 h after treatment with H_2_O_2_, following the completion of apoptotic processes.

Accompanying the activation of the innate immune system in noise-induced cochlear pathologies, HMGB1 might play an essential role as a mediator of inflammation by alarming of the induced cell damage (Tang et al., 2012; Yang et al., 2013; Wood and Zuo, 2017; Frye et al., 2019; He et al., 2020). As a representative DAMP, the biological activity of HMGB1 is known to depend on its location and its interaction with cytokines or other exogenous and endogenous molecules (Andersson and Tracey, 2011). Its dynamic translocation from the nucleus with a state binding DNA to the cytoplasm or extracellular context has been reported to depend on cell activation, injury, or death (Ueda and Yoshida, 2010). In the present study, we investigated the rare high dense extracellular distribution of HMGB1 following NE, even at the local point of absent scattered OHCs. The main reason behind this finding could be the characteristic deposition of HMGB1, which might vary among necrotic and apoptotic cells. Breaking-down cell permeability could facilitate the passive release of HMGB1 from necrotic cells; however, under apoptotic conditions, maintaining nuclear retention of HMGB1 would be necessary for chromatin binding through posttranslational modifications (Scaffidi et al., 2002; Rovere-Querini et al., 2004; Bell et al., 2006; Kazama et al., 2008). Importantly, OHCs are known to be vulnerable to damage induced by excessive noise, with apoptosis appearing to be the most common mechanism of OHC loss (Morrill and He, 2017). Therefore, the release of HMGB1 from the nucleus of OHCs was shown to be highly restricted after acoustic injury. Alternately, a possibility exists that the translocation behavior of HMGB1 could be a temporary process, indicating that more sensitive and intensive detection methodology might be required for the observation of its dynamic distribution (Gaillard et al., 2008). This was consistent with our results here revealing the presence of a high density of HMGB1 in the cytoplasm of HEI-OC1 cells at 8 h that disappeared at 12 h after treatment with H_2_O_2_. Furthermore, we evaluated the changes in the expression level of caspase-3 by western blotting. There was a sharp elevation at 12 h after treatment with H_2_O_2_, indicating an extensive activation of apoptosis, potentially explaining the molecular event of the retention of HMGB1 in the nucleus (Bell et al., 2006).

Maintaining the structural and functional integrity of the basilar membrane in the cochlea, is known to require a highly orchestrated process for the elimination of damaged OHCs (Hu et al., 2018; Li et al., 2018b; Zhou et al., 2020). This process has been shown to always involve the activation of neighbor cells or professional scavengers, such as macrophages. Although the organ of Corti lacks immune cells, the cochlear resident cells, particularly DCs, are known to preserve their phagocytic activity responding to damaged OHCs (Abrashkin et al., 2006; Du et al., 2011; Yang et al., 2015b). This was supported by strong piece of evidence showing that prestin immunopositive fragments of OHCs were located inside the cytoplasm of DCs (Abrashkin et al., 2006). However, the mechanism by which DCs detect the injury of OHCs remains unclear. Ladrech et al. proposed that DCs might regulate the remodeling of the aminoglycoside-injured organ of Corti by releasing HMGB1 (Ladrech et al., 2013). This hypothesis was strongly supported in immunoelectron microscopy experiments, by the detection of HMGB1 particles encapsulated within cytoplasmic membrane-bound vesicles inside DCs. More importantly, these vesicles were labeled as specific markers of processing secretion, indicating the function of DCs to initiatively secrete HMGB1 (Ladrech et al., 2013). In our present study, we found that the density of optical HMGB1 labeling was drastically increased in DCs after acoustic trauma, especially in the 1dPNE group. Overall, the spatiotemporal dynamics of the distribution of HMGB1 in the organ of Corti suggested a specific pattern of immune activity involving DCs, which might exert a direct impact on the pathogenesis of OHCs after acoustic trauma.

Apart from the organ of Corti, the level of HMGB1 was also tested in non-organ of Corti tissues including spiral limbus, spiral ligament, and SGNs. The density of optical labeling of HMGB1 was found to be greatly increased in the spiral ligament, but not SGNs. In the SGN region, the number of glial cells with high level of HMGB1 was demonstrated to be increased in the 3dPNE group, indicating a possible intercellular event between SGN and glial cells. In the spiral ligament, we found that the tendency of AFD elevated (Figures 3C,D), which was consistent with the change in the total cochlear level of HMGB1 (Figure 1B). Necroptosis-, ferroptosis-, apoptosis- and pyroptosis-mediated mechanism have all been implicated in HMGB1 release from cells (Scaffidi et al., 2002; Bell et al., 2006; Murai et al., 2018; Wen et al., 2019; Volchuk et al., 2020). Specifically, as a marker of cellular damage, the increasing HMGB1 level or the release of HMGB1 in the spiral ligament indicated the activation of cell death pathways. In previous studies, noise-induced apoptosis and necroptosis in the spiral ligament cells have been elucidated, but the pyroptosis and ferroptosis have not been verified and might cause the HMGB1 release here (Wang et al., 2019; Wu et al., 2020). Therefore, our future work will focus on defining HMGB1 sources and secretion pathways in this region during noise-induced inflammation.

In the present study, our *in vitro* experiments showed that knocking down the HMGB1 gene could protect HEI-OC1 cells against hydrogen peroxide stress damage (Figure 5), indicating potential therapeutic approaches to prevent NIHL. However, it might not be an optimal approach to manipulate the expression of HMGB1 due to its intracellular functions, such as acting as a critical pro-autophagic protein that enhances cell survival and limits programmed apoptotic cell death (Tang et al., 2010, 2011; New and Thomas, 2019). Moreover, enhancing the autophagic flux has been shown to prevent the cochlea from acoustic injury and reduce the percentage of absent OHCs (Yuan et al., 2015). Therefore, the critical point of any potential therapeutic strategy of NIHL targeting HMGB1 should avoid affecting its positive intracellular functions. Recently, treatments based on antagonists that specifically inhibit the release of HMGB1 or extracellular action of HMGB1 have been released. For example, an anti-HMGB1 monoclonal antibody (mAb) has been developed and was shown to be effective in treating many animal models of central nervous system (CNS) diseases, such as Parkinson’s disease, Alzheimer’s disease, and stroke (Yang et al., 2015b).

Nevertheless, an approach of inhibiting the activities of proinflammatory mediators, such as HMGB1, to treat inflammation-induced diseases might have some considerable challenges: (1) targeting HMGB1 might not be sufficient to obtain significant beneficial results because of homologous molecules playing the same role as HMGB1 in the inflammatory process; (2) triggering a compensatory immune response involving an alternative pathway after inhibiting the extracellular function of HMGB1; (3) risks associated with weakening of a natural defense mechanism in the cochlea (Tabas and Glass, 2013). Furthermore, we still need further *in vivo* experiments to confirm the specific role of HMGB1 in cochlear inflammation in order to fully elucidate the underlying mechanism and be able to intervene to prevent NIHL.

## Data Availability Statement

The original contributions presented in the study are included in the article/supplementary material, further inquiries can be directed to the corresponding author/s.

## Ethics Statement

The animal study was reviewed and approved by the Ethics Committee of the Shanghai Jiao Tong University Affiliated Sixth People’s Hospital.

## Author Contributions

LXiao and YSu conducted the experiment, wrote the manuscript, and analyzed the data. LXia, GY, and YF designed the experiment. CL, ZZ, and YSh interpreted the data. All authors have read and approved the final manuscript.

## Conflict of Interest

The authors declare that the research was conducted in the absence of any commercial or financial relationships that could be construed as a potential conflict of interest.
